# Factors Associated With the Intention to Participate in Coronavirus Disease 2019 Frontline Prevention Activities Among Nursing Students in Vietnam: An Application of the Theory of Planned Behavior

**DOI:** 10.3389/fpubh.2021.699079

**Published:** 2021-07-02

**Authors:** Quynh Anh Tran, Huong Thi Thanh Nguyen, Tung Van Bui, Nguyet Thi Tran, Nguyet Thi Nguyen, Tham Thi Nguyen, Hien Thu Nguyen, Son Hoang Nguyen

**Affiliations:** ^1^School of Preventive Medicine and Public Health, Hanoi Medical University, Hanoi, Vietnam; ^2^College of Health Sciences, VinUniversity, Hanoi, Vietnam; ^3^Hanoi Medical College, Hanoi, Vietnam; ^4^Institute for Global Health Innovations, Duy Tan University, Da Nang, Vietnam; ^5^Faculty of Pharmacy, Duy Tan University, Da Nang, Vietnam; ^6^Center of Excellence in Evidence-based Medicine, Nguyen Tat Thanh University, Ho Chi Minh, Vietnam

**Keywords:** intention, COVID-19, frontline, nursing students, the theory of planned behavior

## Abstract

**Introduction:** Medical students have been serving as a key part of the frontline health workforce responding to the coronavirus disease 2019 (COVID-19) pandemic globally. Their contribution is especially important in the resource-scarce settings of developing nations such as Vietnam. Yet, the intention of medical students, in particular, nursing students, to participate in COVID-19 frontline prevention activities has not been well-understood. This study aimed to examine factors associated with the intentionto participate in COVID-19 frontline prevention activities among Vietnamese nursing students.

**Methods:** A cross-sectional study was conducted on a total of 597 students in December 2020 in Hanoi, Vietnam. Information regarding the socioeconomic characteristics of participants, their source of COVID-19 related knowledge, and their perception and attitude toward participating in COVID-19 frontline activities [based on Theory of Planned Behavior (TPB)] was collected. A hierarchical regression model was employed to examine the association between intentions of students and associated factors.

**Results:** A positive intention to participate in COVID-19 frontline prevention activities was found (mean score of 25.3 over 35; SD = 4.4; min = 5; max = 35). Attitude toward behavior, subjective norms, and perceived behavioral control (PBC) was found to be significantly associated with the intention of students. These variables explained the 37% variation in the intention of students in the model. Among three factors, subjective norm showed the strongest correlation with intention of students (β = 0.358; *p* < 0.001). Obtaining information from official sources and community was also found to be positively correlated with intention to participate.

**Conclusion:** Most of the respondents reported a positive intention to participate in COVID-19 frontline prevention activities. The findings suggested that the TPB was a good instrument to predict the intention to perform behavior among Vietnamese students. Enhancing the positive attitude of students, encouraging family and community supports, and providing adequately essential resources will contribute to optimizing the participation of students to confront COVID-19.

## Introduction

On March 11, 2020, the WHO formally declared the coronavirus disease 2019 (COVID-19) a pandemic (1), reflecting an inability to contain its spread, and as of March 28, 2021, over 126 million confirmed cases, including over 2.7 million deaths, have been reported worldwide (2). Facing the shortage of health workforce as a result of the COVID-19, expanding the health workforce by the recruitment of retirees or final year of healthcare students is a possible solution for many countries. In Denmark, final-year medical students have been employed as temporary residents at hospitals (3). In the United Kingdom, the hospitals have successfully integrated medical students into nursing teams (4). The experiences of the COVID-19 pandemic in Poland have shown that medical students who have worked voluntarily in frontline health services reported a low level of fear and received positive feedback from family, friends, patients, and healthcare workers (5). In the US, medical students can deploy to local health agencies to implement rapid testing, join student outbreak response teams, implement critical preventive policies, or be staff of community call centers that offer guidance and services to individuals with symptoms of or exposed to COVID-19 (6). Recent studies reported factors influencing the intention of medical students to work in the frontline during the COVID-19 pandemic including family support (7), the availability of protective personal equipment, and the risk of infection (8). A study in Denmark revealed that 80% of medical students in a university had decided to join the COVID-19 pandemic frontline healthcare workforce (9).

The first case of COVID-19 in Vietnam was declared on January 23, 2020, and as of March 28, 2021, over 2,500 confirmed cases and 35 deaths were reported (10). Vietnam has been acknowledged as one of the most successful countries in the world in controlling morbidity and mortality due to COVID-19 through the integration of resources from multiple sectors, such as health, mass media, education, public affairs, and defense (11). The Vietnam Ministry of Health has announced to mobilize the entire health workforce for the prevention and control of the COVID-19 pandemic, in which students at healthcare universities and colleges, including medical students, nursing students, or pharmacy students, have to be trained on disease prevention, patient care, surveillance, detection tests, and measures to prevent epidemics in the community. Furthermore, these students should be ready to voluntarily participate in COVID-19 prevention tasks when assigned (12).

**Figure 1:**
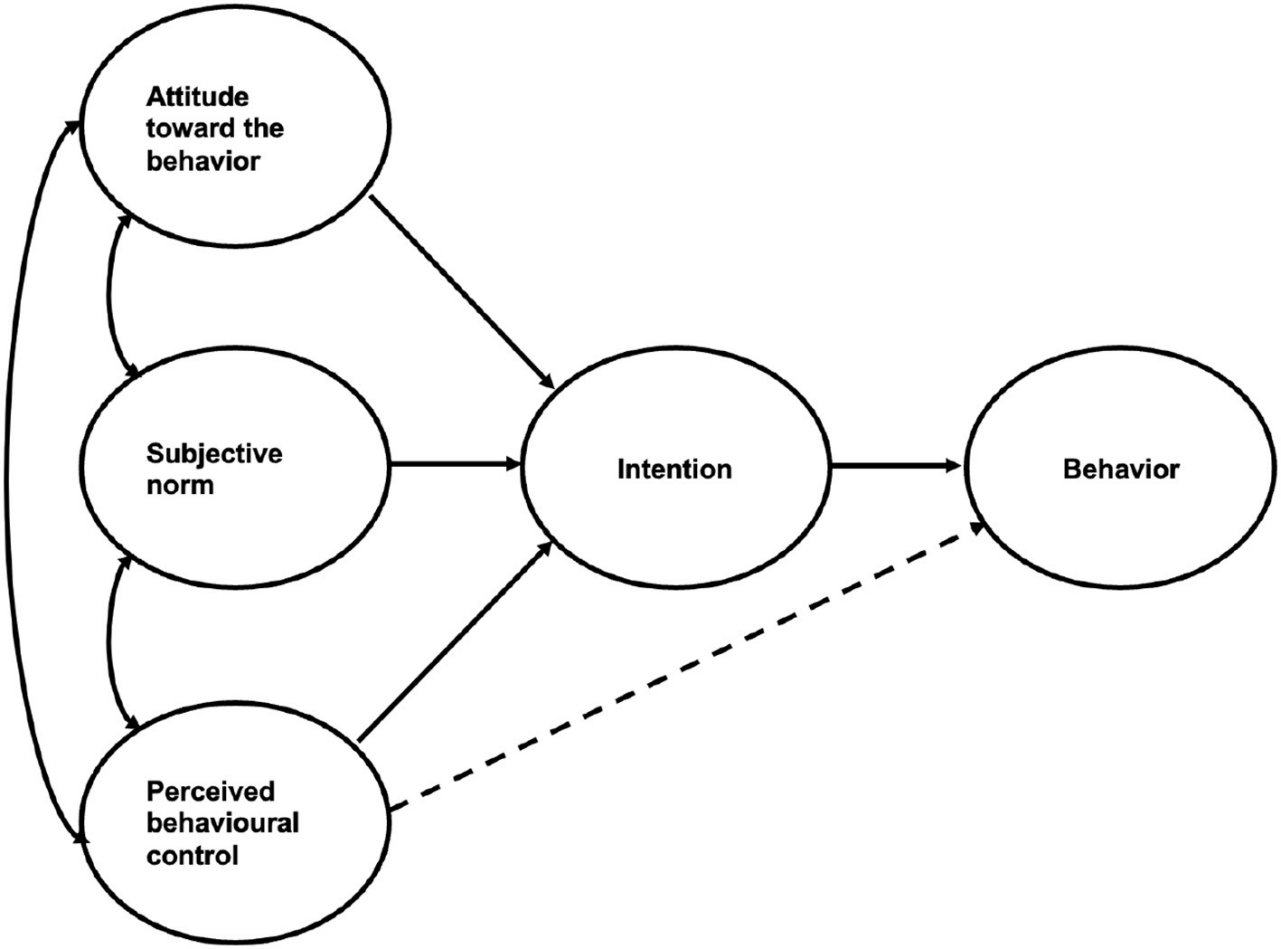
The Theory of Planned Behavior (TPB).

According to the Theory of Planned Behavior (TPB), human behavior could be predicted by the intention to perform that behavior (13). This behavioral intention is determined by three components (Figure 1): attitude toward the behavior (the degree to which a person has a favorable or unfavorable evaluation of the behavior), subjective norm (perception of an individual about the particular behavior, which is influenced by the judgment of significant others such as parents or friends), and perceived behavioral control (PBC) (the perceived ease or difficulty of performing the behavior of interest) (14). The more favorable the attitude and subjective norm, and the greater the perceived control, the stronger should be the intention of the person to perform the behavior (15). This psychological theory was developed by Icek Ajzen to improve the predictive power of the Theory of Reasoned Action by adding the factor of PBC in the model (14). The TPB has been applied in many research worldwide to explain a variety of health-related behaviors, for example, taking HPV vaccination (16), using mental health services (17), or participating in physical activities (18). Furthermore, the TBP has been used to predict the behaviors of health professionals in previous studies, including the intention to care for patients with emerging infectious diseases (19) or to care for patients with severe acute respiratory syndrome (SARS) among nurses in Korea (20) and Taiwan (21). Previous studies also have demonstrated that three factors of TPB explained a 20–50% variation in the intention of the participants to perform study behavior (16, 19, 21, 22).

Although Vietnam has achieved certain success in the fight against the COVID-19 pandemic, the potential risk of new epidemic waves requires the readiness of the entire healthcare workforce, including university and college students. The nursing students are young people trained to be nurses in the future, who care directly for patients in clinical settings. This study aims to examine how TPB factors are associated with the intention to participate in COVID-19 frontline prevention activities among a sample of nursing students in Hanoi, Vietnam.

## Methods

### Study Setting and Participants

A cross-sectional study was conducted on nursing students at the Hanoi Medical College in 2020. The inclusion criteria of participants in this study were as follows: (1) students in the 3-year nursing training program at Hanoi Medical College, (2) students agreeing to participate in the study through the consent form, and (3) students having the full ability to answer the question. Students who reported to be ill or unwell on the survey day were excluded. There have been about 30 classes each year with an estimated number of 25 students per class at the Hanoi Medical College. First, we randomly selected eight classes per year of education and then invited all students available in classrooms on the survey day to participate in the survey. A total of 597 students from the first year to the third year of the nursing training program completed the self-anonymous questionnaires. The response rate was 100%.

### Measurement

The self-anonymous questionnaires have been developed to measure the intention of students and three associated factors based on the guideline of constructing TPB questionnaires (14, 15) and references from previous studies (19–21). The questionnaires were reviewed by three experts in the fields of nursing, behavioral sciences, and public health. Before the main survey, a pilot survey was conducted with 30 students to examine the clarity of the items, the completion of the questionnaires, and the time required. Based on the pilot study and consultation with experts, some changes were made to increase the quality of the questionnaires. All measures use a 7-point Likert scale, ranging from 1 (strongly disagree) to 7 (strongly agree). Negative items were coded reversely before scoring the scales.

The study instrument has been used to collect the following information:

***Socioeconomic characteristics include***age, sex, year of education, residence of the family (urban/rural), having family/relatives working in the health sector (yes/no), having elderly in the family (yes/no), living with parents/relatives (yes/no), and information sources about COVID-19.

The intention of students toward behavior was measured by a scale of five items in which respondents reported their perception of whether they would like to participate in COVID-19 frontline prevention activities in different levels such as be always ready to participate, participate when it is needed, or consider participating. Intention score of each respondent was calculated by the sum of five items. A higher score showed a more positive intention about the behavior. The intention toward behavior scale had a moderate internal consistency (Cronbach's α is 0.64). Three components predicted the intention of students:

***Attitude of students toward participating in COVID-19 frontline prevention activities*** was measured by their belief about the benefits and disadvantages of performing the behavior (e.g., “Participating in COVID-19 frontline prevention activities is a good opportunity for learning by doing,” and “Participating in COVID-19 frontline prevention activities will increase the infection risk for me”). The attitude score of each student was calculated by the sum of eight items on the scale. A higher score indicated a more positive attitude toward behavior. The attitude scale had a relatively high internal consistency (Cronbach's α is 0.76).

***Subjective norm***was measured by the normative beliefs of students in participating in COVID-19 frontline prevention activities by considering the support from family (parents, siblings, neighbors, and community where they are living), school (teachers, school policies), and peers (girlfriends/boyfriends, close friends, and classmates). Individual subjective norm score was calculated by the sum of seven items on the scale. A higher score presented a greater normative belief about the behavior. The subjective norm scale had a high internal consistency (Cronbach's alpha is 0.90).

***Perceived behavioral control***was measured by the confidence of students in their knowledge and skill to perform the behavior. This knowledge and skill refer to the availability of institutional resources, including updated information, personal protective equipment, and assistance from other health professionals. PBC indicates the perception of students of how easy or difficult it would be to participate in COVID-19 frontline prevention activities. Individual PBC score was calculated by the sum of seven items on the scale. A higher score showed greater perceived control of the behavior. The PBC scale had a relatively high internal consistency (Cronbach's α is 0.76).

### Data Analysis

Data were analyzed using STATA version 15. The intention toward behavior was treated like a dependant variable, while attitude, subjective norm, and PBC were treated as independent variables. Descriptive statistics were used to analyze the general characteristics of participants and the mean of the intention scale. The Pearson correlation was applied for scales of the TPB. The hierarchical linear regression was performed to examine the association among three TPB components and the intention of students to participate in COVID-19 frontline prevention activities controlled by general characteristics variables of the students and the sources of knowledge about COVID-19. The associations were tested using α <0.05 as the level of statistical significance.

The formula for a multivariable linear regression is as follows: *Y* = *a* + *b*_1_ * *X*_1_ + *b*_2_ * *X*_2_ + … + *b*_*i*_ * *X*_*i*_,

where *Y* is the dependent variable, *X*_*i*_ is the independent variable, *a* is the intercept, and *b*_*i*_ is the regression coefficient of the variable *X*_*i*_.

We applied the hierarchical linear regression with four models:

Model 1: Students' intention = *a* + Attitude

Model 2: Students' intention = *a* + Attitude + Subjective norm

Model 3: Students' intention = *a* + Attitude + Subjective norm + PBC

Model 4: Students' intention = *a* + Attitude + Subjective norm + PBC + General characteristics of the participants + Sources of knowledge about COVID-19.

## Results

### General Characteristics of the Participants and the Scales

Table 1 shows that, of the 597 students who completed the questionnaires, most were females (79.1%); the average age is 19.6; two-fifth of the students (39.5%) reported family relatives working in the health sector; three-fifth of the students (61.7%) reported having family residence in urban areas; half of the students (50.8%) reported living with their parents/relatives; over a half of the students (57.8%) reported having elderly in the family. Regarding the sources of knowledge about COVID-19; 58.0% of respondents reported that they receive information from the Ministry of Health, 56.0% from the community, and 23.6% from the universities.

**Table 1:** General characteristics of the participants and the intention score (n = 597).

	***n***	**%**	**Intention (ranged score: 1–35)**	
			**Mean ± SD**	***p***
**Gender**				
Male	125	20.9	25.1 ± 4.4	>0.05
Female	472	79.1	25.3 ± 4.6	
**Age (mean** **±** **SD; min; max)**	19.6 ±1.5; 18; 28
**Year of education**				
First-year students	198	33.2	25.8 ± 4.1	>0.05
Second-year students	202	33.8	25.4 ± 3.9	
Third-year students	197	33.0	24.7 ± 5.0	
**Having family relatives work in the health sector**				
Yes	236	39.5	25.5 ± 4.3	>0.05
No	361	60.5	25.1 ± 4.5	
**Family's residence**				
Urban area	362	61.7	25.1 ± 4.5	>0.05
Rural area	225	38.3	25.6 ± 4.3	
**Living with**				
Parents/relatives	303	50.8	25.6 ± 4.0	>0.05
Others	294	49.2	24.9 ± 4.8	
**Elderly people in the family**				
Yes	345	57.8	25.1 ± 4.5	>0.05
No	252	42.2	25.5 ± 4.3	
**Sources of knowledge about COVID-19 (*****A question with many answers*****)**				
Ministry of health	346	58.0	25.9 ± 3.7	<0.01
University/College	141	23.6	26.2 ± 3.9	<0.01
Relatives/friends	109	18.3	26.1 ± 3.9	<0.05
Community	334	56.0	25.4 ± 4.4	>0.05
Others	23	3.9	25.8 ± 4.4	>0.05

Table 2 presents the characteristics of four scales used to measure the intention of the participants and three associated factors. Descriptive statistics show that the average score is relatively high across all assets. Among four scales, three scales measuring independent variables have high internal consistencies and normal distributions. The mean score of the intention to participate in COVID-19 frontline prevention activities was 25.3 of 35, indicating a positive attitude.

**Table 2:** Characteristics of the scales.

**Scale**	**Number of items**	**Mean; SD**	**Min; Max**	**Skewness; Kurtosis**	**Cronbach's α**
Intention	5	25.3 ± 4.4	5–35	−1.2; 5.4	0.64
Attitude	8	38.7 ± 6.2	14–56	−0.01; 3.6	0.76
Subjective norms	7	36.2 ± 8.3	7–49	−0.7; 3.5	0.90
PBC	7	35.7 ± 5.8	11–49	−0.7; 4.2	0.76

Table 3 shows statistically significant positive relationships between the intention to participate in COVID-19 frontline prevention activities and the attitude toward behavior (*r* = 0.361, *p* < 0.01), subjective norm (*r* = 0.542, *p* < 0.01), and PBC (*r* = 0.491, *p* < 0.01). Besides, bivariable correlations among three associated factors were positive and significant.

**Table 3:** Correlations between the scales of the Theory of Planned Behavior (TPB).

	**Intention**	**Attitude**	**Subjective norms**	**PBC**
Intention	1			
Attitude	0.361^*^	1		
Subjective norms	0.542^*^	0.409^*^	1	
PBC	0.491^*^	0.447^*^	0.512^*^	1

* *p < 0.01*;

*Pearson's r-values are presented*.

### Association Between TPB Factors and the Intention of Students

Table 4 presents the relationship between three factors of TPB and the intention of students to participate in COVID-19 frontline prevention activities by hierarchical linear regression analysis. Model one showed that attitude explained a 12.9% variance in the intention of students (β = 0.361, *p* < 0.001). In model two, attitude and subjective norms explained 31.5% variance in the intention of students (β = 0.167 and 0.474, respectively; *p* < 0.001). In model three, three components of TPB explained a 35.9% variance in the intention of students. The last model explained a 37% variance in the intention of students with the entry of background variables and sources of information (*R*^2^ = 0.370; *F* = 23.99, *p* < 0.001). Receiving knowledge of COVID-19 from the Ministry of health (β = 0.146, *p* < 0.01) and community (β = 0.114, *p* < 0.01) were significant variables with model changes.

**Table 4:** Hierarchical linear regression of study variables.

	**Model 1**	**Model 2**	**Model 3**	**Model 4**
**Attitude**	0.361^*^	0.167^*^	0.093^**^	0.083^**^
**Subjective norms**		0.474^*^	0.372^*^	0.358^*^
**Perceived behavioral control**			0.259^*^	0.247^*^
**Gender (vs. female)**				
Male				−0.034
**Year of education (vs. first-year students)**				
Second-year student				−0.017
Third-year student				−0.034
**Having family relatives work in the health sector (vs. no)**				
Yes				0.015
**Family's residence (vs. urban)**				
Rural areas				0.005
**Living with (vs. parents/relative)**				
Other				0.048
**Elderly people in the family (vs. no**)				
Yes				0.019
**Sources of knowledge about COVID-19**				
***Ministry of health (vs. no)***				
Yes				0.146^*^
***University (vs. no)***				
Yes				0.005
***Relatives/friends (vs. no)***				
Yes				−0.020
***Community (vs. no)***				
Yes				0.114^*^
***Others (vs. No)***				
Yes				0.063
***R***^**2**^	0.129	0.315	0.359	0.370
***p***	<0.001	<0.001	<0.001	<0.001
***F***	88.93	137.93	112.16	23.99

** *p < 0.01*,

* *p < 0.05; Standardized coefficients are presented*.

## Discussion

To our knowledge, this is the first study to explore the intention of students to participate in the COVID-19 frontline prevention activities and associated factors based on the TPB in Vietnam. The findings revealed a positive intention to participate in COVID-19 frontline prevention activities among a sample of nursing students in Hanoi (mean score 25.3 of 35). This level of intention is higher than the intention to care for patients with SARS among nurses in Taiwan (21) and Korea (20). This difference explained by the COVID-19 mortality is relatively low in Vietnam compared to other countries in the world (10), while SARS was known as a life-threatening emerging disease with higher mortality (23), and the participation in prevention activities has a lower risk than in caring for patients. However, the mean score of the intention scale in this study is in line with the study on the intention to care for patients with emerging infectious diseases among the sample of Korean nurses (19), suggesting a similarity in risk perception among the two populations.

The TPB was selected as a theoretical framework for this study because it has been used successfully to understand health-related behaviors and other professional behaviors in many countries; however, it has not been applied in Vietnam. With the aim of the study to examine the association between three components of TPB and the intention to participate in the COVID-19 frontline prevention activities, we found that attitude, subjective norm, and PBC were significant factors associated with the intention. Students who reported a more positive attitude, greater perceived support from surrounded people, and stronger PBC were more likely to have greater intention to participate in the COVID-19 frontline prevention activities when needed. The linear regression model including these three factors, controlled by the general characteristics of the respondents, has explained a 37% variance in the intention. Previous studies reported different results. A systematic review and meta-analysis on food safety behavioral intention revealed that the accumulated TPB explained 22% of the total true effect variance (22). Another study found an explanation for the 35% variance in the intention of nurses to care for patients with SARS (21) while this figure increased to 54 and 55.1% on the intention of taking HPV vaccination and the intention of nurses to care for patients with emerging infectious diseases, respectively (16, 19). Collectively, these results suggested the applicability of the TPB in predicting behavioral intention among the general population and health professionals as well.

In this study, the subjective norm was found to be the most significant among predictor variables. Subjective norm, a perception about behavior influenced by the judgment of significant others, was reported as the most influential factor to predict the intention to practice food safety in the US (22). In a study, Feng and Wu (24) explained the influence of Chinese and Taiwanese culture on subjective norms that then influence the behaviors of nurses in caring for patients. Taiwanese people, like many Asian people including Vietnamese, tend to be less individualistic than Western people and so their behaviors are more influenced by the values and points of view of other people (24). However, previous studies on the intention of nurses to care for patients with SARS and patients with emerging infectious diseases reported that PBC was the strongest predictor of intention (19, 21), not the subjective norm. This can be explained that nurses had experiences in their professional field then they understood better about the advantages and disadvantages of the performing behaviour than nursing students. In contrast, nursing students in our study are young people (mean age of 19.6), therefore the support of families, peers, schools, and communities is very important and strongly influenced to their normative beliefs.

The study found that obtaining COVID-19 related information from the Ministry of Health and from the community positively influences the intention of nursing students to participate in frontline prevention work. This provides some insights into the relationship between the source of information regarding COVID-19 and the desire to join the frontline medical workforce of medical students, which has otherwise been under-researched. Existing literature on the intention of medical students to participate in COVID-19 prevention activities has investigated the correlation between other factors and intention to participate. A study on Chinese medical students cited pressure and the extent of support from family as correlating factors (7), while others found sense of purpose (25, 26), desire to help (8, 25), and to learn and gain professional experience (8, 27) to be some of the most common reason for medical students to participate in frontline COVID-19 prevention activities. Knowing the possible influence of source of knowledge about COVID-19 on the intention of participation of nursing students implies that such intention can be encouraged by managing the content of information distributed through official (Ministry of Health) and community channels. It is crucial for students to receive accurate and timely information about the pandemic, particularly on issues that relate to their possible participation in prevention work, including the availability of personal protective equipment, the implementation of necessary procedures at health facilities and communities to identify and isolate possible positive case, and how participation can affect their study and possible compensation.

In the context of the COVID-19 pandemic, the mobilization of all health workforce, including medical students, nursing students, and other healthcare students, is essential to confront the shortage of human resources for both developed and developing countries. Student response teams were initially formed at the country like the US (28) and Vietnam (29) with the participation of a limited number of students and schools. In Vietnam, the capacity of local authority and community on epidemics was identified to be moderate (30). Therefore, to mobilize students effectively and broadly, understanding predictors of the behavioral intention of students will provide evidence for education and communication programs. University teachers play an important role in improving the attitudes of students to participating in COVID-19 frontline prevention activities as good opportunities for learning by doing. Communication programs should encourage family and communities to support the activities of students. Besides, providing adequately essential resources such as protective personal equipment and updated information about the disease is needed to increase the PBC of students.

This study has some limitations. First, it is considered that other important associated factors with students might not be included in this study, for example, knowledge of students about COVID-19 prevention. This weakness should be improved in further research. Second, participants recruited from a college located in Hanoi, the capital of Vietnam, may not represent a majority of students throughout the whole country. Third, the instrument developed for the first time in Vietnamese with limited items needs to be tested further to increase the consistency and reliability of scales. Further studies are needed to overcome these limitations and examine the behavioral changes corresponding to the different stages of the epidemic.

In conclusion, this study found that most of the respondents reported a positive intention to participate in COVID-19 frontline prevention activities. Three components of TBP, namely, attitude toward behavior, subjective norm, and PBC, were significantly associated with the intention of the student, suggesting that the TPB was a good instrument to predict the intention to perform behavior among Vietnamese students. The education program should consider these factors to encourage the participation of students to confront COVID-19.

## Data Availability Statement

The raw data supporting the conclusions of this article will be made available by the authors, without undue reservation.

## Ethics Statement

The studies involving human participants were reviewed and approved by Hanoi Medical University research proposal committee, Hanoi Medical University, Hanoi, Vietnam. The patients/participants provided their written informed consent to participate in this study.

## Author Contributions

QAT, HTTN, NTT, and TTN: conceptualization. HTTN, TVB, NTT, and NTN: data curation and methodology. HTTN, TVB, NTT, TTN, and HTN: formal analysis and writing the original draft. QAT, NTN, and TTN: investigation. QAT, TTN, and SHN: supervision and writing, review, and editing. All authors contributed to the article and approved the submitted version.

## Conflict of Interest

The authors declare that the research was conducted in the absence of any commercial or financial relationships that could be construed as a potential conflict of interest.
